# Chromosomal instability: a key driver in glioma pathogenesis and progression

**DOI:** 10.1186/s40001-024-02043-8

**Published:** 2024-09-04

**Authors:** Adele Mazzoleni, Wireko Andrew Awuah, Vivek Sanker, Hareesha Rishab Bharadwaj, Nicholas Aderinto, Joecelyn Kirani Tan, Helen Ye Rim Huang, Jeisun Poornaselvan, Muhammad Hamza Shah, Oday Atallah, Aya Tawfik, Mohamed Elsayed Abdelmeguid Elsayed Elmanzalawi, Sama Hesham Ghozlan, Toufik Abdul-Rahman, Jeremiah Adepoju Moyondafoluwa, Athanasios Alexiou, Marios Papadakis

**Affiliations:** 1 Barts and the London School of Medicine and Dentistry, London, UK; 2 Sumy State University, Sumy, Ukraine; 3 Department Of Neurosurgery, Trivandrum Medical College, Trivandrum, India; 4 Faculty of Biology Medicine and Health, The University of Manchester, Manchester, UK; 5 Internal Medicine Department, LAUTECH Teaching Hospital, Ogbomoso, Nigeria; 6 Faculty of Medicine, University of St Andrews, St. Andrews, Scotland, UK; 7 Royal College of Surgeons in Ireland, University of Medicine and Health Sciences, Dublin, Ireland; 8 School of Medicine, University College Dublin, Dublin, Ireland; 9 School of Medicine, Queen’s University Belfast, Belfast, UK; 10 Department of Neurosurgery, Hannover Medical School, Carl-Neuberg-Strasse 1, 30625 Hannover, Germany; 11 Faculty of Biotechnology, October University for Modern Sciences and Arts (MSA), Giza, Egypt; 12 Graduate University for Advanced Studies, SOKENDAI, Kanagawa, Japan; 13 Arab Academy for Science, Technology & Maritime Transport, Alexandria, Egypt; 14 Faculty of Clinical Sciences, Department of Medicine and Surgery, Ile-Ife, Osun State Nigeria; 15 University Centre for Research & Development, Chandigarh University, Chandigarh-Ludhiana Highway, Mohali, Punjab India; 16 Funogen, Department of Research & Development, Athens, Greece; 17 Department of Research & Development, AFNP Med, 1030 Vienna, Austria; 18 Department of Science and Engineering, Novel Global Community Educational Foundation, Hebersham, NSW 2770 Australia; 19 Department of Surgery II, University Hospital Witten-Herdecke, University of Witten-Herdecke, Heusnerstrasse 40, 42283 Wuppertal, Germany

**Keywords:** Chromosomal instability, Gliomas, Neuro-oncology, Molecular genetics

## Abstract

Chromosomal instability (CIN) is a pivotal factor in gliomas, contributing to their complexity, progression, and therapeutic challenges. CIN, characterized by frequent genomic alterations during mitosis, leads to genetic abnormalities and impacts cellular functions. This instability results from various factors, including replication errors and toxic compounds. While CIN’s role is well documented in cancers like ovarian cancer, its implications for gliomas are increasingly recognized. CIN influences glioma progression by affecting key oncological pathways, such as tumor suppressor genes (e.g., TP53), oncogenes (e.g., EGFR), and DNA repair mechanisms. It drives tumor evolution, promotes inflammatory signaling, and affects immune interactions, potentially leading to poor clinical outcomes and treatment resistance. This review examines CIN’s impact on gliomas through a narrative approach, analyzing data from PubMed/Medline, EMBASE, the Cochrane Library, and Scopus. It highlights CIN’s role across glioma subtypes, from adult glioblastomas and astrocytomas to pediatric oligodendrogliomas and astrocytomas. Key findings include CIN’s effect on tumor heterogeneity and its potential as a biomarker for early detection and monitoring. Emerging therapies targeting CIN, such as those modulating tumor mutation burden and DNA damage response pathways, show promise but face challenges. The review underscores the need for integrated therapeutic strategies and improved bioinformatics tools like CINdex to advance understanding and treatment of gliomas. Future research should focus on combining CIN-targeted therapies with immune modulation and personalized medicine to enhance patient outcomes.

## Introduction

Chromosomal instability (CIN) is a significant biological phenomenon involved in the etiology of many illnesses, most notably cancer [1]. CIN is characterized by an increased frequency of genomic changes during mitotic processes, disrupting the genetic content distribution to progeny cells. This results in the accumulation of genetic abnormalities, which significantly impacts cellular performance. CIN has become a more widely recognized and essential determinant in carcinogenesis and disease progression in cancer genetics [2].

The consistency of chromosomal preservation and distribution during mitotic division is critical to cellular life and performance. Nonetheless, genomic integrity is constantly threatened by various harmful agents, including replication errors, exposure to toxic compounds, and endogenous reactive oxygen species [3]. Cellular systems have evolved reparative processes to repair DNA damage, bolstering genetic integrity. However, breaches in these protective regimes can result in CIN, which manifests in various genomic repercussions, including aneuploidy, chromosomal translocations, and genomic amplifications, all of which have carcinogenic implications.

CIN has been linked to several cancers, most notably ovarian cancer [4]. Emerging evidence, however, links CIN to brain malignancies, specifically gliomas, which are glial progenitor-derived neoplasms [5]. Elucidating the link between CIN and gliomas may provide unique molecular insights, leading to novel diagnostics and treatments. Despite advances in clinical therapy, gliomas continue to have poor prognosis due to their proliferative activity and resistance to traditional medicines.

Recent findings have highlighted the role of non-coding RNAs (ncRNAs) in modulating CIN, adding another layer of complexity to the regulation of genomic stability. DNA methylation-related long non-coding RNAs (DMlncRNAs) modulate gene expression by interacting with chromosomal modifications or remodeling factors, thereby affecting genomic instability and glioma progression. In lower grade gliomas (LGGs), specific DMlncRNAs have been identified as key regulators of genome instability and the tumor microenvironment (TME), impacting immune cell infiltration and patient prognosis [6]. This underscores the importance of epigenetic regulation in gliomagenesis and the potential for DMlncRNAs to serve as prognostic markers and therapeutic targets. This highlights the interplay between genetic and epigenetic mechanisms in gliomagenesis.

CIN-mediated changes commonly intersect critical oncological pathways, affecting key tumor suppressor genes, oncogenes, and DNA repair-centric genes such as TP53, PTEN, and EGFR [7–9]. The cumulative genomic landscape created by CIN gives neoplastic cells an adaptive advantage, allowing them to avoid canonical regulatory checkpoints and sustain uncontrolled proliferation [10]. In gliomas with pronounced CIN, the interplay between canonical and non-canonical DNA repair pathways becomes particularly evident. For example, homologous recombination deficiency (HRD), characterized by an impaired ability to repair double-stranded breaks through homologous recombination repair (HRR), can exacerbate genomic instability and tumor progression [11]. Tumors with HRD often exhibit heightened sensitivity to interstrand crosslink (ICL)-inducing therapies and poly(ADP-ribose) polymerase (PARP) inhibitors. Notably, traditional methods for assessing HRD, such as chromosomal microarray (CMA), may not capture the full spectrum of HRD signatures compared to advanced techniques like optical genome mapping (OGM), which can detect additional variants indicative of HRD [11]. This enhanced sensitivity underscores the need to explore both canonical and non-canonical repair mechanisms to fully understand how CIN drives glioma progression and influences treatment outcomes. Gliomas with significant CIN are associated with increased virulence, correlating with poorer clinical outcomes [10].

As CIN continuously modifies the genomic constitution of neoplastic cells, emergent sub-clonal entities have the potential to survive existing treatment options [12]. CIN, a hallmark of human cancer, results from errors in chromosome segregation during mitosis, leading to structural and numerical chromosomal abnormalities. This not only generates genomic heterogeneity that acts as a substrate for natural selection but also promotes inflammatory signaling by introducing double-stranded DNA into the cytosol, engaging the cGAS-STING anti-viral pathway [12]. These multipronged effects highlight CIN as a central driver of tumor evolution and underscore its role in the interaction between tumor cells and the microenvironment, influencing immune editing and evasion. Understanding the genetic complexities of gliomas and CIN’s influence on them, particularly in treatment-resistant glioma subtypes and their responses to currently available interventions, is crucial.

Recent discoveries of the interaction of CIN and gliomas can reshape oncological care, potentially opening the door to precision-oriented therapy options. Nonetheless, a complete understanding of CIN’s roles in gliomas and its interplays with further pathological mechanisms currently needs to be improved.

## Methods

A narrative review was carried out to provide a comprehensive overview of CIN in all documented types of gliomas. Articles restricted to the English language were included from inception until August 2024. Pubmed/Medline, EMBASE, the Cochrane Library and Scopus databases were searched, including the terms “chromosomal instability” “glioma,” and their respective synonyms. To ensure the completeness of the review, the reference lists of included articles were manually searched for additional relevant studies. References cited in recent reviews on similar topics were also manually reviewed to identify additional sources that could contribute to the search strategy. Standalone abstracts, conference proceedings, case reports, and posters were excluded, with priority given to the inclusion of high-quality and reliable evidence. In addition, the review did not limit the number of studies to provide a comprehensive understanding. It included descriptive, animal model, cohort, and observational studies from both preclinical and clinical settings to provide a holistic perspective. Both the adult and pediatric populations were also included in order to ensure a comprehensive summary of all the currently available literature on the topic up to date. Table 1 summarizes the methodology.

**Table 1:** Summary of methodology

Methodology steps	Description
Literature search	PubMed/MEDLINE, EMBASE, Scopus and the Cochrane Library
Inclusion criteria	Various study designs including experimental studies, randomized controlled trials, prospective and retrospective cohort studies Studies involving both pediatric and adult populations Studies providing raw data Full-text articles published in English
Exclusion criteria	Non-English studies, stand-alone abstracts, conference proceedings, editorials, commentaries, and letters
Search terms	Key words such as ‘chromosomal instability’, ‘CIN’, and ‘Gliomas’’ were used for a comprehensive database search
Additional search	A manual search was performed to include references from recently published procedure-specific and disease-specific reviews
Sample size requirement	No strict sample size requirement

## Genetic background of CIN and an overview of CIN in early glioma development and progression

CIN is a well-established form of genomic instability with enduring relevance in cancer research. Over a century ago, pioneering observations by Theodor Boveri [13] and David von Hansemann [14] unveiled the presence of structural and numerical chromosomal aberrations as defining characteristics of cancer. CIN originates from genetic mutations affecting genes responsible for maintaining chromosomal structure and governing mitotic processes. This disruptive mechanism yields substantial chromosomal damage, afflicting chromosome count and structural integrity [15, 16]. CIN leads to various outcomes, including segmental ane­uploidy, point mutations, CNAs, and structural modifications. The impact is so significant that it can result in the acquisition or loss of chromosomal se­gments or even e­ntire chromosomes within a single mitotic e­vent [17]. It is important to emphasize that although structural chromosomal modifications and aneuploidy can be indicative—hallmarks of CIN, they do not always represent the same underlying processes [18]. In specific clinical contexts and congenital conditions such as trisomy 21, aneuploidy can remain static or stable, decoupled from the dynamic CIN process, further highlighting the intricate spectrum of genomic instability [19–21]. CIN encapsulates the continuum of chromosomal alterations across successive cellular generations, portraying a multifaceted facet of cancer biology.

The emergence of micronuclei typically concomitant with CIN highlights a significant cellular consequence of this instability. During cell division, chromosomes that fail to integrate into a daughter nucleus give rise to micronuclei encapsulated within their distinct nuclear membranes [22]. In the context of gliomas, the primary cell line NCH149, characterized by elevated levels of numerical CIN, exhibits spontaneous micronucleus formation, albeit accompanied by a relatively modest degree of re CIN [23, 24]. Notably, passages of NCH149 cells manifested a substantial prevalence of micronuclei, reaching proportions of 33% and 71% respectively, underscoring the dynamic nature of this process [23, 24]. Moreover, the size of chromosomes exhibits a discernible correlation with the propensity to become sequestered within micronuclei [25]. Chromosomes featuring larger kinetochores have been demonstrated to be more prone to forming erroneous merotelic kinetochore–microtubule attachments, with reported error rates of 7.0% compared to 1.6% for chromosomes endowed with smaller kinetochores, as evidenced in studies involving Indian muntjac cells [26]. This phenomenon may be attributed to chromosomal lagging from kinetochore size discrepancies and potential cohesion fatigue. It is plausible that larger chromosomes are more susceptible to cohesion fatigue, thereby elevating the likelihood of merotelic attachments, further perpetuating the cycle of CIN in these cells.

Chromosomes that find themselves enclosed within micronuclei in cancer cells are susceptible to a catastrophic phenomenon known as chromothripsis. This intricate process has been documented in more than 500 cases cataloged in a meticulously curated chromothripsis database, which raises intriguing questions regarding the potential preferential damage to larger chromosomes by chromothripsis in specific tumor types and human malignancies as a whole [22, 27–29]. A wealth of studies has elucidated the pivotal role of centrosome amplification in fostering CIN within tumor cells [30–32]. Within animal cells, centrosomes occupy a central position in microtubule organization [33]. These centrosomes are instrumental in orchestrating the formation of bipolar mitotic spindles during mitosis, a requisite step for precisely segregating chromosomes [34]. The duplication of centrosomes is a highly regulated event, typically occurring at the onset of S-phase entry, ensuring that each daughter cell inherits a single centrosome during cytokinesis. Consequently, a cell may possess either one unduplicated centrosome or two duplicated centrosomes, with maintaining centrosome number homeostasis subject to stringent control mechanisms. Centrosome amplification, characterized by abnormal mitotic spindle formation and an elevated incidence of errors in chromosomal segregation, arises when this regulatory process becomes dysregulated [33, 34].

By directly influencing the DNA damage response (DDR) activity, including the DNA damage checkpoint and the DNA repair machinery, the genetic changes that characterize glioma genomes may also be responsible for the suboptimal treatment response of such tumor types. Cells activate the phosphoinositide 3-kinase (PI3KK)-related kinases ATM, ATR, and DNA-dependent protein kinase (DNA-PK) in response to DNA damage. Following on, these kinases phosphorylate numerous downstream substrates, including the effector kinases Chk1 and Chk2, leading to the initiation of cell-cycle checkpoints and apoptosis. Several other proteins known as checkpoint mediators or adaptors, such as 53BP1, BRCA1, and MDC1, are also necessary to activate DDR signaling. Recent research has suggested that the DDR functions as a checkpoint for tumor growth, requiring early malignant lesions to inactivate p53 or other DDR components to advance to more aggressive stages [35].

In glioblastoma patients, copy number loss of the genes encoding the ATM/Chk2 and ATR/Chk1 pathways frequently occurs, with heterozygous loss of CHEK2 being the most common occurrence. The RCAS/tv-a system, in conjunction with platelet-derived growth factor (PDGF)-induced glioma models, has served as a valuable platform for investigating critical DNA Damage Response (DDR) molecules in glioma development. This experimental approach has shed light on the pivotal roles of ATM, Chk2, and p53, demonstrating their indispensability in restraining glioma tumor progression in murine models. Notably, the loss of any of these genes accelerates tumor growth and imparts a more aggressive phenotype to the gliomas, thereby elevating the incidence of high-grade tumors. Furthermore, the absence of Chk2 in gliomas results in compromised cell cycle checkpoints and apoptotic responses. Intriguingly, this deficiency undermines the survival benefits typically conferred by ionizing radiation (IR) observed in control mice [36, 37].

Recent findings have highlighted the potential of variations within the telomere domain to instigate genetic diseases, foster genomic diversity, and promote cell immortalization [38]. Critical telomere shortening leads to telomere malfunction, followed by successive bridge-fusion-breakage cycles, and results in numerical chromosomal abnormalities [39, 40]. In cells with critical telomere dysfunction, dicentric chromosome formation, and genomic instability, there is an increased vulnerability to oncogenic transformation [41–43]. Such findings have been consistently replicated, showing that telomerase activity is frequently found in malignant cerebral tumors, including glioblastoma, and is linked to shorter telomeres [44, 45]. The findings suggest that telomerase activity and shorter telomeres could indicate human glioma malignancy.

**Fig. 1:**
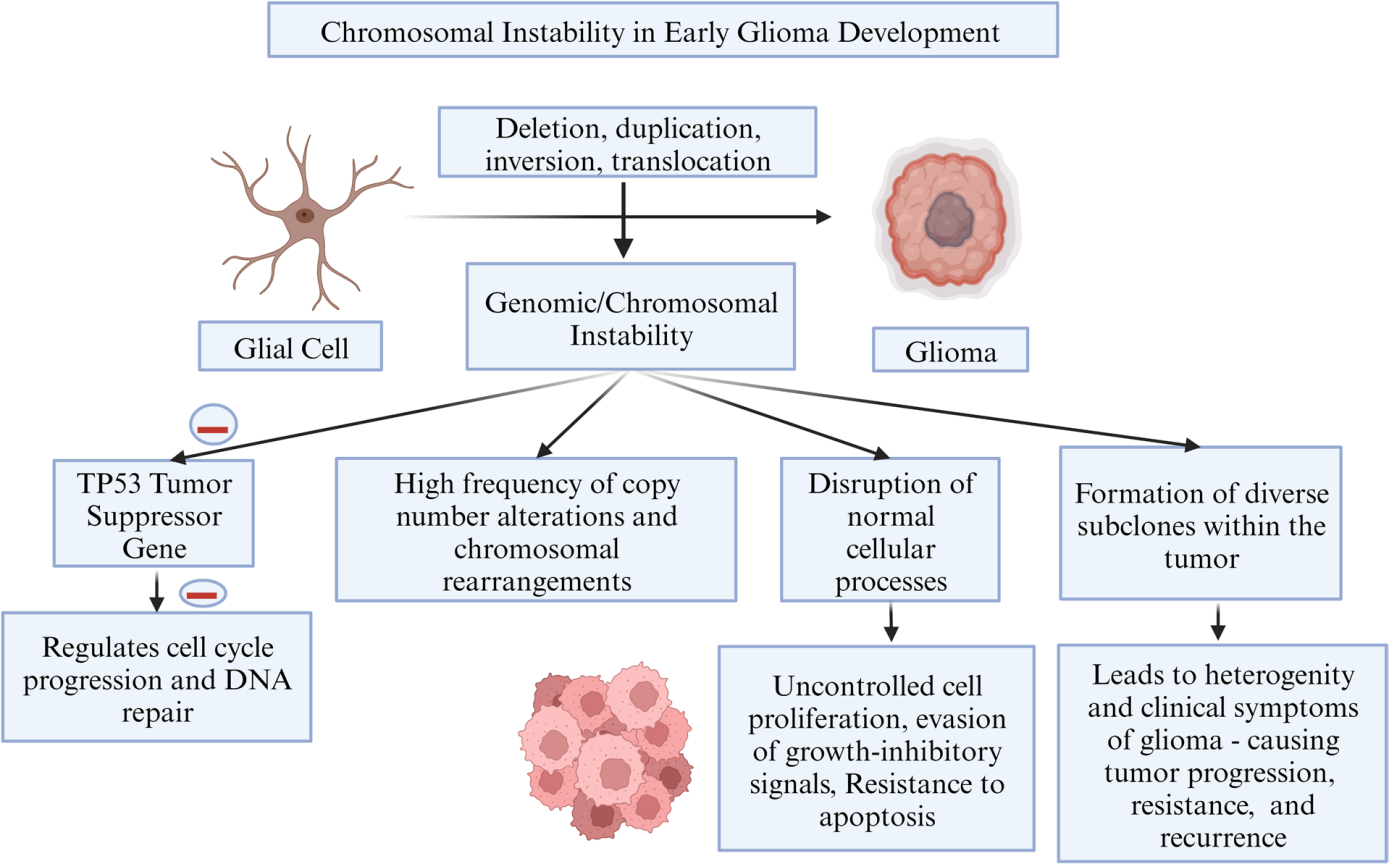
Role of chromosomal instability in the development of early glioma. DNA: Deoxyribonucleic acid

A significant link between supernumerary centrosomes and cancer, particularly about aberrant chromosomal segregation, is well established [46]. This theory has spurred extensive investigations into the role of supernumerary centrosomes in various types of cancer. An early study employed electron microscopy to explore the presence of supernumerary centrosomes in gliomas [47]. The results revealed both the existence of some supernumerary centrioles and centriole clusters. This research laid the groundwork for further investigations into the role of centrosomes in gliomas. Indeed, subsequent studies have consistently observed the prevalence of supernumerary centrosomes in high-grade glioma samples, particularly within prominent nuclei [48, 49]. Centrosome-specific antibodies were used to identify supernumerary centrosomes, and these findings have aligned with similar observations made in other cancer types. These collective findings underline the significance of supernumerary centrosomes in cancer development and their potential impact on chromosomal segregation. Compared to normal tissue, gliomas showed indicators of mitotic dysregulation, such as supernumerary centrosomes. Numerous malignancies frequently have elevated quantities of mitotic regulatory proteins and changed levels of centrosome structural proteins; hence, therapies that target these protein classes may soon be developed [50–52]. The role of CIN in early glioma development is summarized in Fig. 1.

## Chromosomal instability and gliomas subtypes

### Adult gliomas

#### Glioblastoma—IDH wildtype

**Fig. 2:**
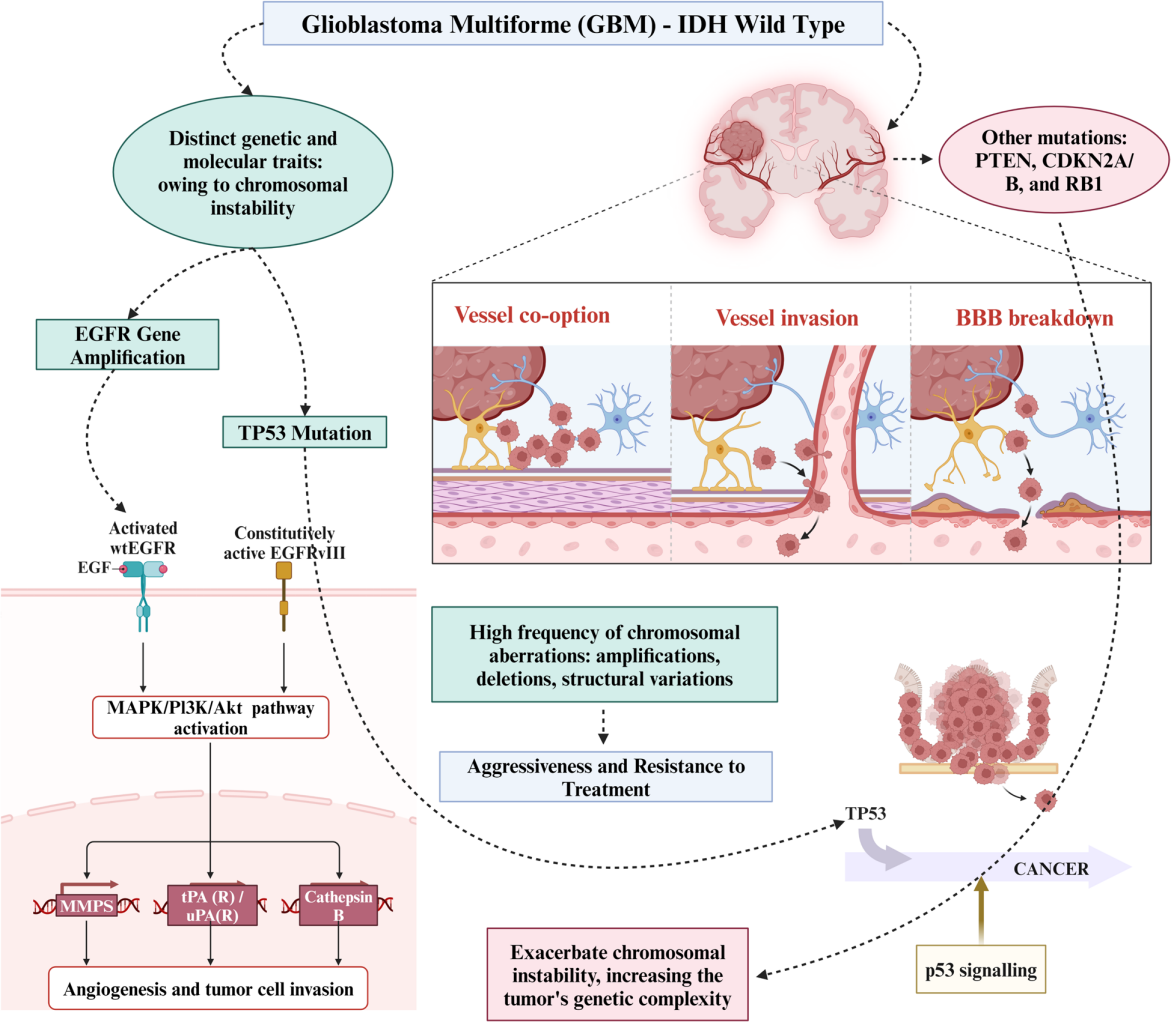
Role of chromosomal aberrations in the progression of glioblastoma—IDH wildtype. glioblastoma multiforme, GBM; isocitrate dehydrogenase, IDH; epidermal growth factor receptor, EGFR; tumor protein p53, TP53; phosphatase and tensin homolog, PTEN; cyclin-dependent kinase inhibitor 2A, CDKN2A; retinoblastoma protein 1, RB1

Glioblastoma is a highly aggressive brain tumor with a dismal prognosis [53]. The “IDH wildtype” subtype of glioblastoma stands out due to specific genetic and molecular characteristics and its particularly pronounced CIN [54]. The genomic profiling of glioblastoma tumors has revealed a significant occurrence of chromosomal aberrations, such as amplifications, deletions, and structural variations [55]. The IDH wildtype subtype, which comprises the majority of glioblastoma, lacks mutations in the isocitrate de­hydrogenase (IDH) gene [56]. These tumors exhibit heightened CIN and frequently display structural and numerical chromosomal abnormalities [56]. This instability is a hallmark of IDH wildtype glioblastoma and substantially influences their aggressive behavior and resistance to therapeutic interventions [54]. CIN within the context of glioblastoma has exhibited a propensity to generate genetically diverse subclones, profoundly influencing responses to therapeutic interventions and disease recurrence [57]. Furthermore, augmented CIN within tumor-initiating cells [TICs] has been established to significantly enhance their tumorigenic potential, establishing a direct connection between genomic instability and their intrinsic capacity [58]. In IDH wildtype glioblastoma, one of the pivotal genetic alterations closely associated with CIN is the TP53 mutation, which occurs with notable frequency [59]. TP53, functioning as a tumor suppressor gene, is commonly mutated within this glioblastoma subtype, impairing its conventional role in cell cycle regulation and DNA repair mechanisms [60]. Consequently, genetic mutations accumulate within the tumor cells. In addition, amplifying the epidermal growth factor receptor (EGFR) gene represents another pivotal genetic modification linked to CIN in IDH wildtype glioblastoma [61]. EGFR amplification stimulates uncontrolled signaling pathways within the tumor cells, facilitating their unbridled proliferation and contributing to the genetic tumultuousness characterizing these tumors [61]. Beyond TP53 and EGFR, IDH wildtype glioblastoma frequently exhibit a spectrum of additional genomic alterations, affecting genes such as PTEN, CDKN2A/B, and RB1 [62]. These supplementary genetic anomalies further intensify CIN within the tumor, thereby augmenting the genetic intricacy of these malignancies. Importantly, it is essential to recognize that CIN within IDH wildtype glioblastoma operates through intricate feedback loops, wherein genetic aberrations in one signaling pathway can precipitate dysregulation in others, thus perpetuating a self-reinforcing cycle of genomic instability [63]. The role of CIN in the progression of glioblastoma—IDH wildtype has been illustrated in Fig. 2.

#### Astrocytomas with IDH mutations

**Fig. 3:**
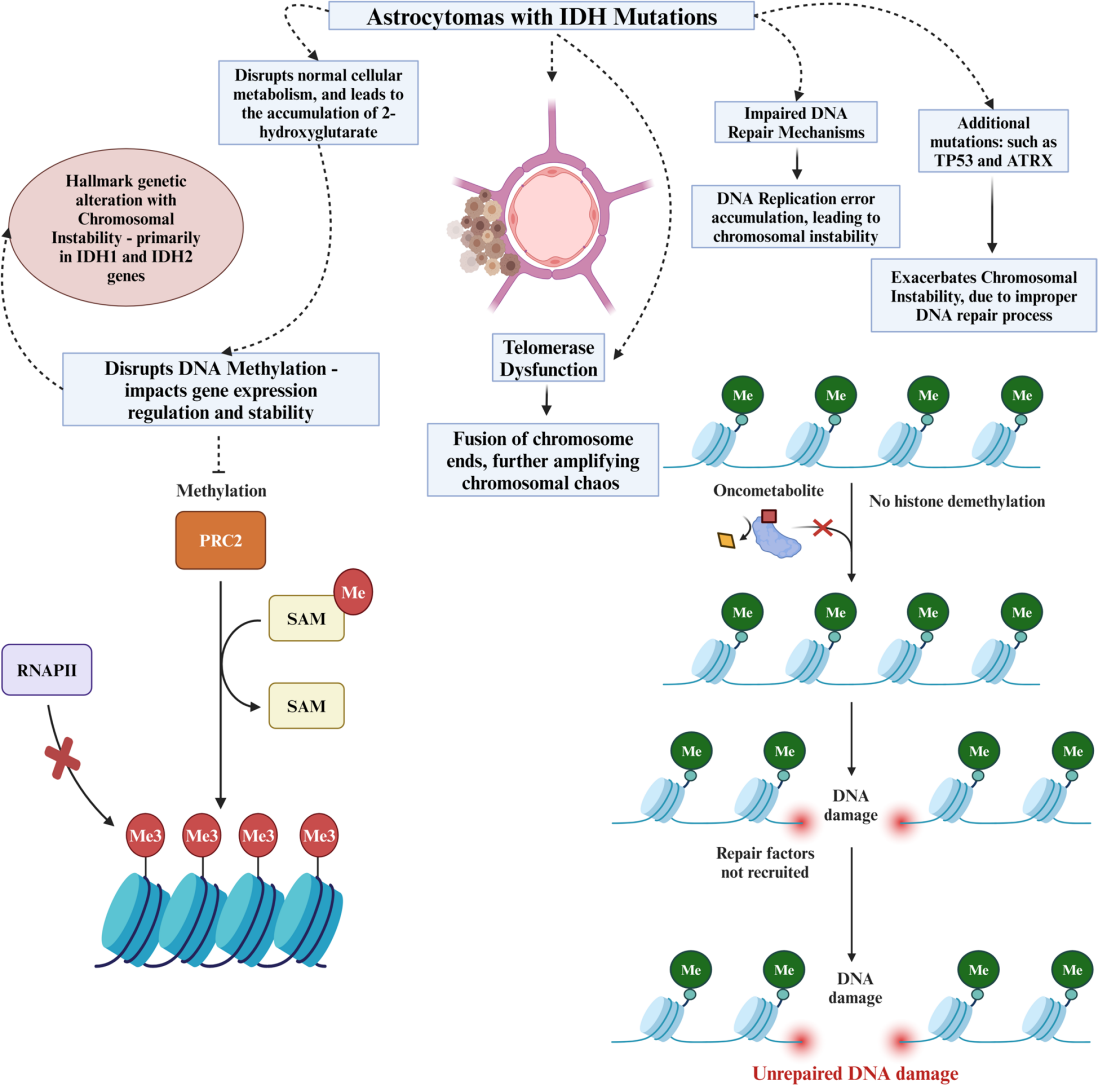
Role of genetic dysregulation in astrocytomas with IDH mutation. IDH 1,2: isocitrate dehydrogenase 1,2. DNA: deoxyribonucleic acid. SAM: S-adenosine methionine. ATRX: ATRX (alpha-thalassemia/mental retardation, X-linked). TP 53: transformation-related protein 53

Astrocytomas demonstrate a diverse genetic landscape that significantly impacts their clinical behavior and outcomes [64]. Within this category of tumors, there is a distinct subgroup characterized by IDH gene mutations, which are associated with CIN [64]. Notably, IDH1 and IDH2 gene mutations serve as a hallmark genetic alteration in astrocytomas linked to CIN [65]. Consequently, the aggregation of these mutations disrupts normal cellular metabolism, resulting in the accumulation called 2-hydroxyglutarate (d-2-HG) [66]. The innate mechanism d-2-HG interferes with DNA methylation, which is indispensable for gene expression regulation and stability and promotes CIN [66, 67]. In parallel, astrocytomas carrying IDH mutations manifest impaired DNA repair mechanisms [68]. The compromised DNA repair apparatus exhibits reduced effectiveness in rectifying DNA damage, including breaks and mutations, resulting in the accrual of errors during DNA replication and repair procedures, thereby contributing to chromosomal instability [68]. Astrocytomas harboring IDH mutations frequently encounter telomere dysfunction, heightening the susceptibility of chromosomes to instability [69]. Telomere shortening and dysfunction can culminate in the fusion of chromosome ends, further amplifying the chaos of chromosomal instability. Additional genetic modifications impacting pivotal genes involved in cell cycle regulation, such as TP53 and ATRX, have been discerned [70]. The dysregulation of these genes exacerbates CIN by perturbing the proper processes of DNA replication and repair. The genetic dysregulation in astrocytomas with IDH mutation has been summarized in Fig. 3.

#### IDH-mutant oligodendroglioma

**Fig. 4:**
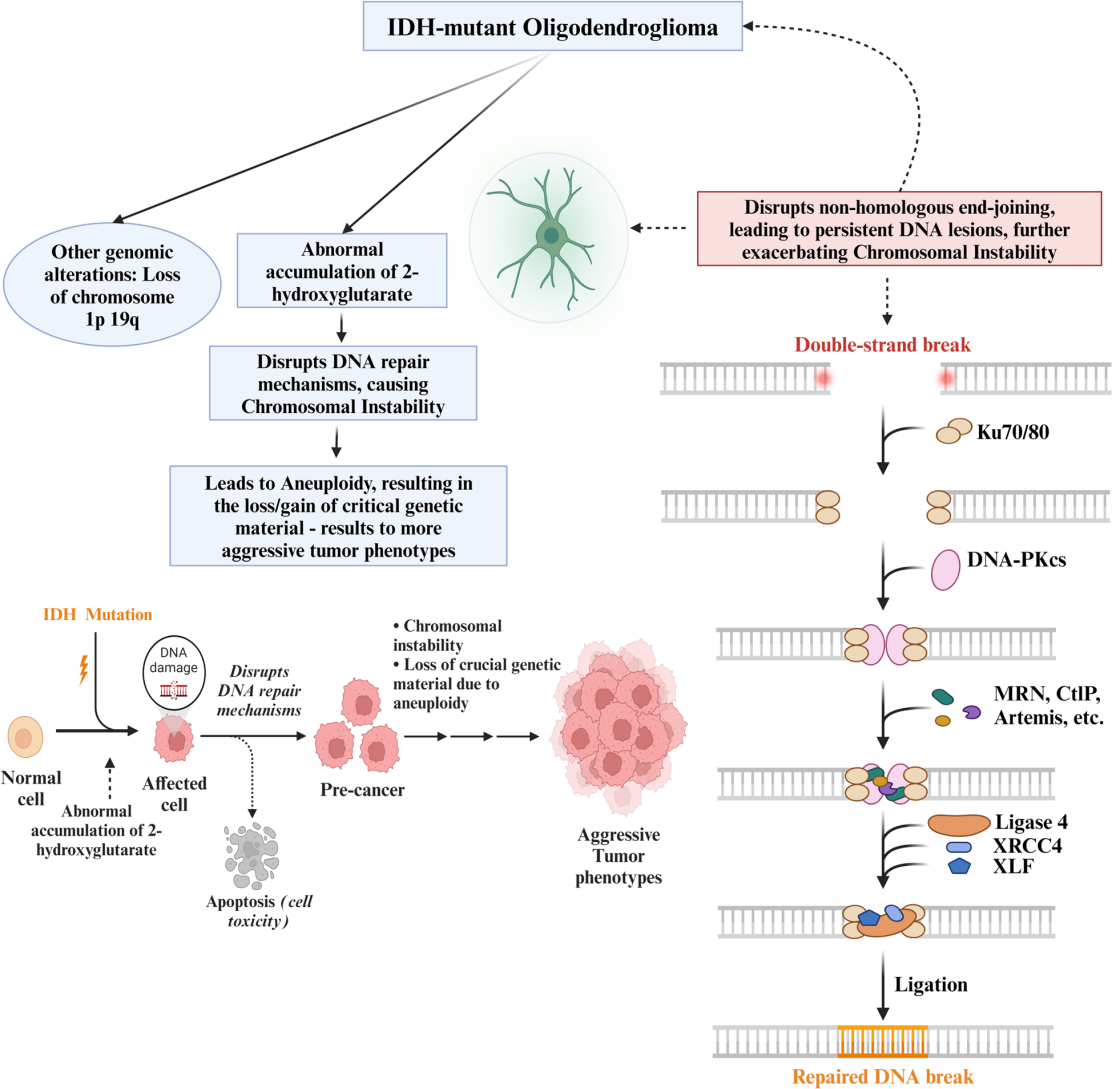
Role of chromosomal instability (CIN) in the pathogenesis of IDH-mutant oligodendroglioma. DNA: deoxyribonucleic acid. IDH: isocitrate dehydrogenase

CIN plays a crucial role in developing and progression of IDH-mutant oligodendrogliomas, unique brain tumors. These tumors are characterized by specific genetic changes involving IDH mutations [71]. As a result of these mutations, an oncome­tabolite called 2-hydroxyglutarate (2-HG) accumulates abnormally [71]. This metabolite disrupts various cellular processes, including DNA repair mechanisms, ultimately contributing significantly to chromosomal instability [72]. The buildup of 2-HG in cells with IDH mutations hinders DNA repair mechanisms such as homologous recombination and non-homologous end-joining. Consequently, it leads to the persistence of DNA lesions and further exacerbates chromosomal instability [72]. CIN in IDH-mutant oligodendrogliomas often leads to aneuploidy, which refers to the loss or gain of critical genetic mate­rial. This process drives the development of more aggressive tumor phenotypes [73]. In particular, specific genomic alterations, such as losses of chromosomes 1p and 19q, regularly occur in IDH-mutant oligodendrogliomas and are closely linked to chromosomal instability [74]. CIN in the pathogenesis of IDH-mutant oligodendroglioma has been summarized in Fig. 4.

### Pediatric gliomas

#### BRAF V660E mutant oligodendroglioma

CIN is central in deciphering the intricate pathophysiology of BRAF V660E mutant oligodendrogliomas, especially in adolescents and children [73, 75–77]. Recent investigations have unveiled a unique subset of tumors within this category, characterized by their IDH wildtype status, oligodendroglioma-like morphology, and BRAF p.V600E mutation [73, 75–77]. Genome-wide methylation of these tumors exhibits a distinctive pattern of CIN, marked by gains in whole chromosome 7 and losses in entire chromosome 10, setting them apart from conventional oligodendrogliomas [73]. CIN, characterized by heightened rates of chromosomal changes encompassing copy number alterations, aneuploidy, and structural variations, plays a pivotal role in developing and progressing these neoplasms [77]. The widespread copy number alterations observed in this subset of tumors, involving more than ten chromosomes, underscore the profound genetic instability that underlies their pathophysiology [73]. Such chromosomal instabilities have been shown to cause aberrant DNA repair mechanisms, impaired cell cycle regulation, and dysregulated signaling pathways, fostering the growth and survival of oligodendroglioma cells [73, 75–78].

Furthermore, the BRAF p.V600E mutation in these tumors is critical to their pathophysiology [78]. This mutation, coupled with the observed CIN patterns, underscores the intricate interplay between genetic alterations and CIN [75]. The coexistence of these molecular features not only differentiates these tumors from conventional oligodendrogliomas but also necessitates precise diagnostic tools to distinguish them from molecular glioblastoma [76]. Understanding the pathophysiology of BRAF V660E mutant oligodendrogliomas within the context of CIN and molecular genetics is paramount for informed clinical decision-making. It enables tailoring treatment strategies to the specific characteristics of these tumors, avoiding unnecessary aggressive interventions when a more indolent clinical course is anticipated [73, 75].

#### Desmoplastic infantile gangliogliomas and astrocytomas

Desmoplastic infantile gangliogliomas (DIGs) and desmoplastic infantile astrocytomas (DAGs) are interesting entities defined within the World Health Organization’s classification of central nervous system (CNS) neoplasms. Such neoplasms typically affect infants and young children, with a predilection for infants under 24 months of age [79, 80]. The characterizations of these tumors are their large size, nodular contrast enhancement, cellular pleomorphism, and undifferentiated small-cell components. Complete surgical resection typically results in an excellent prognosis [80, 81]. Histologically, DIGs and DAGs exhibit similar desmoplastic features: a dense stromal matrix containing fibroblastic and neuroepithelial elements. Neoplastic cells are noted to be confined to solid nodules and adjacent leptomeninges [80]. A deeper analysis of published literature reveals that attempts at analyzing the influence of CINs on DIGs and DAGs have shown inconsistent findings. Comparative genomic hybridization (CGH) and array-based genomic profiling have been used to assess CIN in DIGs and DAGs. According to different studies, these tumors can be attributed to varying chromosomal alterations and no well-defined set of recurrent abnormalities [79–85]. The reporting of chromosomal aberrations has also been inconsistent, with different studies reporting various gains and losses of chromosomal regions [80, 86]. While the precise role of CIN in the pathogenesis of DIG remains unclear, this could influence tumor behavior, including growth patterns and responses to treatment. Still, future studies exploring this is paramount to ascertain definite conclusions.

#### Pediatric astrocytoma

Astrocytomas, the most prevalent type of glial tumors in the pediatric population, have a significant link to CIN [87]. Understanding the implications of CIN is crucial for comprehending the development and progression of these tumors. While genetic abnormalities in specific subtypes like pilocytic astrocytomas have been extensively studied, there is still much to learn about CIN within the broader spectrum of pediatric astrocytomas [88]. Pediatric astrocytomas exhibit considerable genetic chaos with widespread chromosomal imbalances that significantly affect their functionality and biology. The consequences resulting from such are diverse. Morphological plasticity, reflected in their variable histological features, can be attributed to a more significant number of dosage variations in their genes. First, CIN contributes to tumor heterogeneity, leading to the coexistence of genetically distinct subpopulations of astrocytomas within a single tumor [88–91].

Despite the overall genetic complexity, specific subtypes of astrocytomas, such as PXA and SEGA, exhibit a consistent pattern of ge­nomic instability in their subtelomeric rearrangements. Their patterns suggest the presence of specific genomic hotspots that are prone to instability and may contain key driver genes involved in tumorigenesis. A study by Grau et al. (2009) highlights this finding [89]. In addition, CIN is often associated with increased tumor aggressiveness and a higher risk of recurrence. It can drive the progression from low-grade astrocytomas to more malignant forms by promoting the acquisition of genetic alterations seen in higher grade tumors. References [88–90] have all provided evidence supporting this correlation. Specific chromosomal alterations may make patients less re­sponsive to traditional treatments like radiotherapy or chemotherapy. This further limits the options for their treatment.

**Fig. 5:**
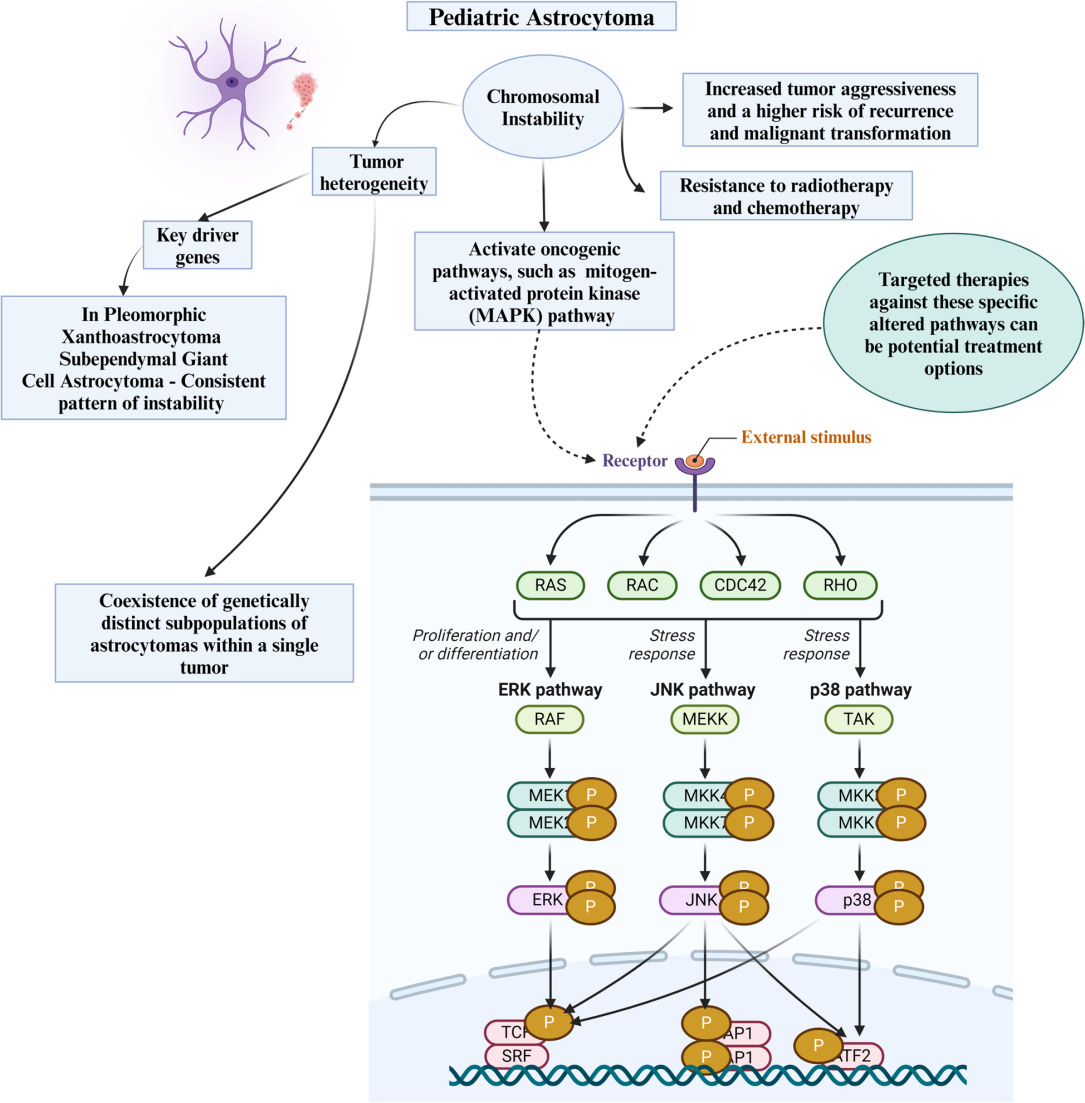
Role of chromosomal instability in pediatric astrocytoma. ERK, extracellular signal regulated kinase; JNK, c-Jun N-terminal kinase; MEK, Mitogen-activated protein kinase kinase

In addition, these alterations can activate oncogenic pathways, such as the commonly disrupted mitoge­n-activated protein kinase (MAPK) pathway in astrocytomas [88]. As a result, targeted therapies that target the specific altered pathways are emerging as promising treatment approaches. This highlights the importance of comprehensive identification and characterization of chromosomal alterations (Fig. 5).

## Association between CIN and immune responses in gliomas

CIN in gliomas is associated with a range of immune effects, including the modulation of immune cell infiltration, antigen presentation, and immune evasion [6, 92]. For instance, CIN-induced genomic alterations can lead to the expression of neoantigens, which may enhance tumor visibility to the immune system [92]. However, the high level of genetic diversity and heterogeneity driven by CIN can also contribute to immune escape mechanisms, such as the upregulation of immune checkpoint proteins and the development of an immunosuppressive TME [10, 35].

In gliomas, CIN often leads to increased levels of tumor-infiltrating lymphocytes (TILs), including T cells and macrophages, which may have varying impacts on tumor progression and treatment responses [93]. For example, elevated levels of TILs have been linked to both positive and negative outcomes in glioma patients, depending on the specific immune cell types and their functional states [93]. CIN also affects antigen presentation by disrupting the major histocompatibility complex (MHC) pathways, potentially impairing the ability of immune cells to recognize and attack tumor cells [35, 36].

Comparatively, in ovarian cancer, CIN similarly influences immune responses by driving the formation of a complex tumor microenvironment with high immune cell infiltration and altered antigen presentation [94]. Ovarian tumors with high CIN often exhibit a heightened immune checkpoint expression, contributing to immune evasion and resistance to immune checkpoint inhibitors [94]. This contrast highlights how CIN can have both analogous and distinct effects on immune responses across different cancer types.

The interplay between CIN and immune responses underscores the need for integrated therapeutic strategies that combine CIN-targeted therapies with immune modulation. Understanding how CIN shapes the immune landscape in gliomas and other cancers can lead to more effective treatments that address both genomic instability and immune evasion, ultimately improving patient outcomes.

## CINdex: a bioinformatics tool for estimating CIN in *cancer* from next-generation sequencing data

DNA copy number change is a critical structural variation in human genomes. CNA refers to changes in copy number resulting from tumor tissue, whereas copy number variations (CNVs) pertain to differences in the copy number of germline cells. Numerous events in the occurrence or severity of malignancies have been linked to the chromosome instability suggested by these CNAs in the DNA. Different technologies, such as genotyping arrays, microarray-based comparative genome hybridization (array CGH), or, more recently, high-resolution next-generation sequencing (NGS), can assess these variations in DNA copy number [95].

Patients’ segment data can be globally analyzed using the Bioconductor software CINdex. Any segmentation method can submit its segment information to the CINdex Bioconductor program. By providing a comprehensive perspective of genomic instability, it calculates a novel measure of genomic instability throughout a chromosome (known as Chromosome-CIN, Standard-CIN, or Regular-CIN) and across cytobands (known as Cytoband-CIN), providing a better resolution of genomic instability.

The CIN values are calculated at the chromosomal and cytoband levels of the CINdex package, standard sections across the whole human genome. This makes it possible to compare the values of chromosomal instability between different patient groups (control vs. case), a common use case in translational research. The software also enables further downstream systems biology studies by linking the differently altered cytobands or chromosomes to genes and pathways [96, 97]. The inception of CINdex is instrumental, serving as an invaluable resource in predicting patient survival outcomes and tailoring personalized treatment paradigms aligned with the distinct genomic attributes of their tumors [98]. The tool’s contribution is eminent in the enhanced comprehension of CIN in gliomas, illuminating the genetic intricacies of the disease and its repercussions on tumor heterogeneity, therapy responsiveness, and overall patient outcomes.

## Limitations of current approaches for CIN evaluation

Evaluating CIN in gliomas is crucial for understanding tumor behavior, prognosis, and response to treatment. Several methodologies are employed to assess CIN, but they come with limitations. For example, comparative genomic hybridization (CGH) compares the DNA of tumor cells to normal cells, detecting gains and losses of chromosomal regions across the entire genome [79–85]. However, CGH has lower resolution and cannot detect balanced translocations or small mutations. In addition, next-generation sequencing (NGS), which involves sequencing the entire genome or exome to provide comprehensive data on mutations, copy number variations, and other genomic alterations [95], is associated with high costs, complex data analysis, and interpretation challenges. To overcome these limitations, future research should focus on developing cost-effective, high-resolution techniques with robust bioinformatics support. Integrating multiple methodologies may provide a more comprehensive assessment of CIN, enhancing the accuracy of diagnosis and the effectiveness of personalized treatment strategies.

CINdex, while valuable for estimating CIN from NGS data, has several limitations. Its reliance on high-resolution segment data [95], which may be prone to inaccuracies or noise, can impact the precision of CIN measurements. The tool primarily measures CIN at chromosomal and cytoband levels [95], potentially missing finer-scale variations. In addition, CINdex’s performance depends on the quality of input data and segmentation methods, which can lead to inconsistencies across studies. Moreover, CINdex does not directly assess the functional consequences of CIN on tumor biology or treatment response, necessitating integration with other analytical methods to fully understand its clinical implications.

## CIN and therapeutic implications

Gliomas present significant challenges regarding prognosis and treatment [99]. Among the hallmarks of gliomas is CIN, a critical factor in their progression [99]. While traditional treatments such as surgery, radiation, and chemotherapy remain foundational, their limitations have spurred interest in innovative therapies specifically targeting CIN [100]. Emerging therapies aim to address the broad spectrum of genetic and molecular aberrations linked to CIN, rather than focusing solely on specific mutations [101].

Targeted therapies have begun to explore the broader implications of CIN. For example, therapies that modulate the tumor mutation burden (TMB), which is often elevated in CIN-driven tumors, could enhance treatment efficacy. Elevated TMB can lead to increased neoantigen presentation, potentially making tumors more susceptible to immune checkpoint inhibitors. Agents such as nivolumab and pembrolizumab, which target immune checkpoints, show promise in this context, though response rates vary among patients [102]. In addition, CAR-T cell therapy, while still experimental for gliomas, could be tailored to target the diverse neoantigens resulting from CIN [103].

Moreover, targeted inhibition of pathways frequently disrupted by CIN, such as the PI3K/AKT/mTOR pathway, offers another strategy. Inhibitors like temsirolimus, which disrupt this signaling cascade, may mitigate tumor growth by countering the downstream effects of CIN [104]. Gene editing technologies like CRISPR/Cas9 also present potential, allowing precise alterations of genes affected by CIN to correct or disrupt their function [105]. However, these approaches face challenges, including the development of resistance, the genetic and phenotypic diversity of gliomas, and the need for personalized treatment strategies.

To effectively target CIN, future research must focus on integrating these approaches, considering how CIN impacts tumor mutation burden and immune response. This broader focus will enhance our ability to exploit CIN for therapeutic benefit, ultimately improving treatment outcomes for glioma patients [103].

## Discussions and prospects

The exploration of CIN in gliomas is paving the way for transformative future perspectives that have the potential to revolutionize our understanding and management of these aggressive brain tumors. Several interconnected areas of research and innovation are poised to shape the landscape of glioma studies.

One prominent avenue is the ongoing investigation into the genomic landscape of gliomas, primarily influenced by CIN. As research advances, we anticipate identifying novel therapeutic targets intricately linked with CIN patterns [106]. This discovery promises to usher in an era of personalized treatments tailored to address the unique genetic anomalies in each patient’s tumor. Such precision medicine approaches hold the promise of significantly enhancing treatment outcomes. Emerging therapeutic strategies are increasingly focusing on CIN-specific vulnerabilities. For instance, agents that modulate microtubules, which play a crucial role in cell division and are affected by CIN, are being explored. In addition, targeting the DNA damage response pathways that are often dysfunctional in tumors with high levels of CIN could provide another therapeutic avenue. In colorectal carcinoma research, high CIN cases have shown that specific chromosomal aberrations, such as the loss of 17p, can occur earlier in cytogenetic evolution, influencing tumor behavior and metastasis [106]. Applying this knowledge to gliomas, identifying similar early and late chromosomal events could guide the development of combination therapies.

The issue of intratumoral heterogeneity, a direct result of CIN, necessitates a shift toward combination therapies as a standard practice in glioma treatment [107]. These multifaceted treatment regimens may encompass agents modulating CIN, like those affecting microtubules, alongside targeted drugs aimed at specific genetic alterations. Success in this realm hinges on our ability to decipher the complex interactions between different genetic subclones coexisting within these tumors.

Another intriguing prospect lies in harnessing CIN as a potential biomarker for early glioma detection and monitoring [108]. In the years to come, refined techniques may enable us to assess CIN within tumor tissue and in easily accessible tissues like peripheral blood lymphocytes. The potential could revolutionize early disease detection and risk assessment, fundamentally altering our approach to intervening in the nascent stages of glioma development. Understanding how CIN contributes to tumor heterogeneity and evolution is crucial. Recent large-scale, high-throughput sequencing studies have highlighted significant genetic diversity within individual tumors [109]. This diversity consists of various subpopulations or subclones that differ spatially and temporally. CIN plays a key role in driving this clonal diversification, working alongside other genetic mechanisms to create the complex genomic instability often observed in cancer. Cancer cells adeptly adjust chromosome missegregation rates to manage the acquisition of genetic diversity while maintaining beneficial genotypes, a strategy that could potentially be leveraged for therapeutic purposes [109]. For instance, whole-genome doubling events can accelerate clonal evolution in certain tumors, leading to favorable near-triploid karyotypes. This suggests that CIN-driven clonal speciation might bypass the reliance on initial truncal mutations [109]. Thus, unraveling the mechanisms governing the transition from advantageous to detrimental effects of CIN on tumor growth represents an intriguing research frontier. A deeper understanding of the precise point at which heightened genomic instability becomes detrimental could open novel avenues for intervention, allowing for controlled induction of CIN in a therapeutic context.

Furthermore, integrating data from diverse omics disciplines, spanning genomics, transcriptomics, and epigenomics, will provide a comprehensive understanding of the intricate relationships underpinning CIN, genetic alterations, and treatment responses. Advanced computational methodologies will play a pivotal role in deciphering the complex molecular interplay within gliomas. The transition from preclinical research to clinical trials is imminent. Robust clinical validation of therapies designed to modulate CIN and rigorous patient stratification based on CIN levels will be pivotal in evaluating safety and efficacy. Ultimately, future perspectives in glioma research will converge on patient-centered approaches. The vision of understanding each patient’s tumor’s unique genomic signature and tailoring treatment plans is rapidly gaining momentum. This paradigm shift toward personalized medicine holds the potential to improve overall patient outcomes significantly.

In summary, the study of CIN in gliomas heralds a new era of precision medicine and innovative therapeutic strategies. While challenges persist, the ongoing synergy between research and technological advancements is poised to shape the future of glioma diagnosis and treatment.

## Conclusion

The study of CIN in gliomas gives crucial information about these complex brain tumors’ origin, progression, and therapy. CIN, characterized by structural and numerical chromosomal irregularities, contributes significantly to the stunning heterogeneity seen in gliomas in both adult and pediatric populations, forming unique genetic profiles within glioma subtypes. CIN’s developing function as a significant biomarker for early detection, risk assessment, and continuous disease monitoring represents an exciting path for investigation. Precision medicine techniques have the potential to revolutionize glioma therapy by tailoring treatments to the precise genomic aberrations caused by CIN, providing hope for better patient outcomes. The study of CIN in gliomas improves our understanding of the genomic intricacies inherent in these tumors and reveals novel therapeutic methods. As we enter the era of precision medicine, the future promises the promise of improved diagnostics, focused therapies, and, ultimately, a better prognosis for those with gliomas.

## Data Availability

No datasets were generated or analysed during the current study.
